# Development of Metabolic Indicators of Burn Injury: Very Low Density Lipoprotein (VLDL) and Acetoacetate Are Highly Correlated to Severity of Burn Injury in Rats

**DOI:** 10.3390/metabo2030458

**Published:** 2012-07-16

**Authors:** Maria-Louisa Izamis, Korkut Uygun, Nripen S. Sharma, Basak Uygun, Martin L. Yarmush, Francois Berthiaume

**Affiliations:** 1 Center for Engineering in Medicine, Massachusetts General Hospital, Harvard Medical School and the Shriners Hospitals for Children, Boston, MA 02114, USA; 2 Department of Biomedical Engineering, Rutgers University, Piscataway, NJ 08854, USA

**Keywords:** hypermetabolism, metabonomics, flux analysis, cluster analysis, regression, variable selection, burn injury

## Abstract

Hypermetabolism is a significant sequela to severe trauma such as burns, as well as critical illnesses such as cancer. It persists in parallel to, or beyond, the original pathology for many months as an often-fatal comorbidity. Currently, diagnosis is based solely on clinical observations of increased energy expenditure, severe muscle wasting and progressive organ dysfunction. In order to identify the minimum number of necessary variables, and to develop a rat model of burn injury-induced hypermetabolism, we utilized data mining approaches to identify the metabolic variables that strongly correlate to the severity of injury. A clustering-based algorithm was introduced into a regression model of the extent of burn injury. As a result, a neural network model which employs VLDL and acetoacetate levels was demonstrated to predict the extent of burn injury with 88% accuracy in the rat model. The physiological importance of the identified variables in the context of hypermetabolism, and necessary steps in extension of this preliminary model to a clinically utilizable index of severity of burn injury are outlined.

## 1. Introduction

Hypermetabolism is a significant consequence of severe trauma such as burns [1], as well as critical illnesses such as cancer [2]. It persists in parallel to, or beyond, the original pathology for many months as an often-fatal comorbidity. Prolonged hypermetabolism is characterized by increased resting energy expenditure and severe muscle wasting due to a negative nitrogen balance. The underlying mechanisms that control the onset and resolution of the hypermetabolic response are unknown. Therefore, current treatments are directed at symptoms which are metabolic, endocrine, and immune in nature.

Increasing nutritional energy delivery and protein intake only partially alleviate the loss of lean body mass [3,4]. Experimental approaches to overcome the deleterious effects of hypermetabolism have been used with varying success, including glutamine and arginine supplementation [5,6]; combinatorial nutritional therapies using a diet high in vitamins, protein, amino acids, and ω-3 fatty acids [7]; peroxisome proliferator activated receptor-α agonists to improve fat oxidation and mitochondrial activity [8]; as well as antioxidant and anti-inflammatory agents [9]. Modulation of insulin action [10], direct insulin therapy [11,12], administration of other anabolic agents [13], as well as β-blockers [14] have also produced significant improvements, but are inherently impractical in the long-term, and in some cases produce undesirable, potentially fatal, side effects [15,16].

Rational design and optimization of nutritional therapies can be achieved by targeting the interconnected metabolic network and regulatory pathways impacted by hypermetabolism [17,18,19,20,21], thereby having a broad impact on the metabolome, and subsequently altering the physiome at the genomic and proteomic levels. Hence an understanding of the connections among individual metabolites and the overall injury physiome is necessary to rationally design metabolic interventions. While the advances in metabolomics and metabonomics are starting to present an ever-increasing amount of data, the capability to quantitatively and accurately predict the metabolic effects of nutritional supplements in order to design the kind of combination therapies that could treat hypermetabolism and other metabolic conditions remains absent.

There are a variety of techniques to extract knowledge from “omics” data. These include pattern identification methods such as clustering and principal component analysis (PCA) typically applied to time-series mRNA microarray data to identify key trendlines [22]; network analysis to identify correlations between genes/metabolites [17]; and marker discovery [23], which utilizes a combination of techniques above to correlate disease with a specific measured variable. Metabonomics in particular focuses on the metabolic analysis of the consequences of a perturbation, such as disease and medication. While metabonomics of toxicity [24] and pharmaceuticals [25] is an emerging field, there are few studies performing rigorous metabolic analyses as a function of extent of injury or disease [23,26].

While there has been some effort to develop analytical mathematical models for hypermetabolism [27,28], predictive models remain absent. There are however efforts to develop such approaches, for instance Flux Balance Analysis (FBA), to identify the metabolic causes of fat accumulation in hepatocytes [29,30]; similar approaches could potentially predict metabolic responses to potential interventions, such as amino acid supplements and hence rationally develop combination therapies in hypermetabolism. However, in vivo hypermetabolism remains a mostly clinical observation, and quantitative measures of hypermetabolism to define the extent of burn injury are necessary for use of optimization-based FBA models.

The objective of this study was to identify sensitive indicators that correlate with the severity of injury and that can be measured from blood samples. For this purpose, we used rat models of cutaneous burn injury of increasing size, and analyzed metabonomic data on postburn day 4. We identified VLDL and acetoacetate in the circulation as being particularly sensitive to the extent of burn injury, and thus could serve as a quantitative measure of the grade of hypermetabolism.

## 2. Experimental Methods

Briefly, male Sprague–Dawley rats (Charles River Labs) weighing between 270 g and 300 g at time of burn, were subjected to a third degree cutaneous burn injury (*i.e.*, depth of injury spans the entire thickness of the skin) covering 20% of the Total Body Surface Area (TBSA) (dorsal burn only) or 40% TBSA (burn on dorsum and abdomen) by contacting the skin with water at 100 °C. Sham-treated animals were handled identically to the burn groups, except that room temperature water was used. After injury, animals were immediately resuscitated with an intraperitoneal saline injection (20 mL/kg per rat) and allowed to recover in individual cages. Animals were weighed daily and food consumption was monitored. On the third day following the injury, all rats were fasted overnight in preparation for the blood samples to be taken on Day 4 as described in detail elsewhere [21]. Briefly, on day 4, each rat was anesthetized and blood flow through each of the major blood vessels entering the liver (the portal vein, PV, and hepatic artery, HA, USA) were measured using a perivascular ultrasonic flow-probe (Transonic Systems, Ithaca, NY, USA). The sum of flow rates into the liver was assumed to equal the flow rate out of the hepatic veins into the suprahepatic vena cava (SHVC). Following flow rate measurements, blood samples from the hepatic veins and PV were taken, followed by arterial blood. Blood samples were analyzed for blood gases and pH using a Rapidlab Blood Gas Analyzer 865 (Bayer). Standard reagent kits were used to determine plasma glucose (Stanbio No. 1075-825), urea (Stanbio No. 0580), and lactate (Trinity Biotech USA No. 735-10). The ketone bodies acetoacetate and β-hydroxybutyrate were measured enzymatically by following the appearance or disappearance of NADH upon the addition of β-hydroxybutyrate dehydrogenase (Sigma), respectively. Nineteen of the common amino acids (except tryptophan) plus ornithine and ammonia were measured using a Waters HPLC apparatus (Waters Co. Milford, MA, USA). ELISA techniques were used to detect albumin (Sigma) and insulin (Crystal Chem Inc No. INSKR020). Alkaline phosphatase (ALP), alanine transaminase (ALT), aspartate aminotransferase (AST), total bilirubin, blood urea nitrogen, and creatinine, HDL, LDL, VLDL, cholesterol and triacylglycerols were measured using a Piccolo Comprehensive Metabolic Panel (Abaxis, Inc., Union City, CA, USA). Finally, the liver was excised and weighed. Fluxes across the liver were subsequently determined as the difference between in- and effluxes, calculated per vessel as the product of metabolic concentration and flow rate normalized to the weight of the liver. Note that early injury markers, such as cytokines and tissue damage, have shown a clear linear trend with increasing burn severity [31,32]; however, we sought variables that were less likely to have a transient response or a response subsequently complicated by sepsis so we only employed metabolic markers still visible 4 days after injury for consideration as indicators of burn injury.

## 3. Numerical Methods

### 3.1. Theoretical Aspects

The primary goal of this study was to identify key metabolites that are strongly correlated to the hypermetabolism in the form of a predictive model of the degree of burn injury. However, since metabolic data suffer from the existence of a high degree of correlation [17], a straightforward statistical analysis (e.g., ANOVA), is very limited. Many variables will be identified as correlated to injury because of their own interrelationships. Therefore, a critical issue is to identify the *key* variables involved, which may be defined as the minimum number of variables that can explain the metabolic response to injury. For the purposes of this work, this problem can equivalently be stated as the identification of the minimum number of variables necessary to construct an accurate model that can predict the degree of injury from metabolic data.

**Figure 1:**
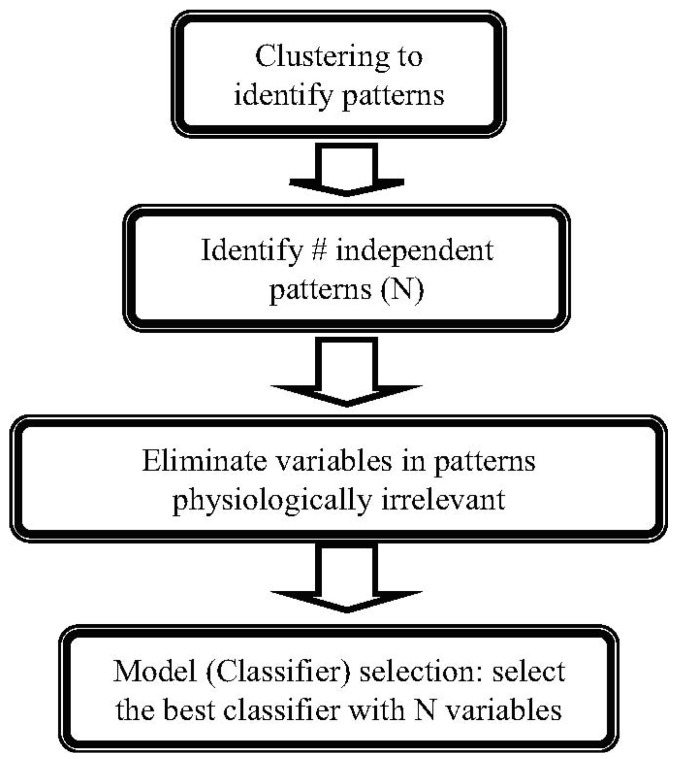
The burn-injury indicator identification algorithm.

It should be noted that in the absence of a mechanistic description of the effects of injury, empirical mathematical models are necessary, which further increases the problem of complexity as the type of model to be used becomes another variable. Therefore, the problem of constructing an “index of burn injury severity” is a multiobjective problem where the task is to simultaneously: (i) select and train the best mathematical model; (ii) maximize model accuracy by selecting the variables to use; (iii) minimize the number of variables in the model. To achieve this goal, we designed a novel algorithm to identify the metabolites that are most indicative of injury grade, as outlined in Figure 1 and discussed in detail below. 

*Clustering of Dose-Response Patterns*. The first step in the algorithm is clustering in order to group the metabolites responding similarly to increased injury, which serves multiple purposes. Clustering serves as the first step in reducing the problem dimensionality, as all metabolites collected in one group can be considered to be regulated by the same mechanisms, and ideally can be considered as a single variable (or, a single metabolite can adequately represent all others in the same cluster). Therefore, the number of clusters necessary to represent the data presents an adequate first-guess for the ideal number of variables to capture the entire metabolic response. In addition, since we have obtained dose-response data rather than a simple injury vs. non-injury comparison, clustering also identifies the major patterns observed in response to increasing levels of burn injury, which enables incorporating a physiological interpretation prior to identification of the burn-grade indicators. 

A critical issue in clustering is the determination of the number of clusters, either defined directly as a parameter (as in k-means clustering which is employed here) or indirectly (as in hierarchical clustering). We utilized a tandem approach to determine the optimum number of clusters, combining analysis of explained variance (here measured as sums of point-to-centroid distances of all clusters) and the separation of individual clusters (assessed through silhouette analysis) [33]. It should be noted that the tandem use is necessary: in clustering, by definition, as the number of clusters increases, the error of clustering is reduced, until each instance (*i.e.*, patient) itself is a cluster and the error is zero, which obviously is of little value. An alternative criterion to identify a good number of clusters that is commonly employed is the marginal gain of adding a cluster, which often displays “elbows” where the marginal value decreases steeply. However, often there are multiple elbows and so multiple choices of good numbers of clusters. To differentiate between such potential solutions, we employed the silhouette analysis, which is a measure of separation for clusters. Briefly, the silhouette value is a measure of how close each point in one cluster is to points in the neighboring clusters. This measure ranges from +1, indicating points that are very distant from neighboring clusters, through 0, indicating points that are not distinctly in one cluster or another, to −1, indicating points that are probably assigned to the wrong cluster [33]. Combining the marginal value and silhouette methods, it is possible to assess marginal return and mean silhouette values as a function of number of clusters, and identify the number of clusters that simultaneously have a local maxima in their silhouette value and display a decrease in cluster error. This is in essence a visual analysis of the two charts to identify the minimum number of clusters 

*Identification of Independent Patterns*. Identified patterns reveal the key trends in the dose response. In mRNA analysis, the co-regulated gene expression reveals information regarding genetic control motifs [34]. The situation is more complex in metabonomics, as correlations exist due to a variety of reasons such as simple stoichiometric dependencies [17]. Accordingly, it is necessary to extract the actual number of independent patterns in order to filter out these metabolic correlations that are not of primary interest for this study.

To achieve this purpose, a Singular Value Decomposition (SVD) was performed on the cluster centroids: Briefly, each cluster was expressed as a vector (the cluster median for each level of injury was an entry in the vector), and the vectors were augmented to form a cluster centroid matrix. SVD was performed to identify the number of independent patterns, which can be used to explain the remaining patterns. This is in effect a rank analysis, but direct analysis of singular values enables identification of marginally non-zero singular values, which are likely artifacts of measurement error and/or statistical clustering rather than major patterns in the injury response. The number of distinct singular values that are also non zero (>10^−2^, a heuristically chosen limit) was chosen as the number of independent clusters.

The product of this tandem approach is: (i) identified patterns in the injury response; and (ii) the minimum number of patterns that can explain the entire response, which is interpreted here as equivalent to the minimum number of variables that are necessary to construct an index of injury.

It is worth noting that this two-step hybrid procedure to reduce the problem dimensionality has problem-specific advantages over conventional dimension reduction methods such as PCA or Linear Discriminant Analysis (LDA). PCA is the optimum method for elimination of collinearities present in the data, but is not optimized for class separability, hence will be insensitive to the presence of patterns due to elevated injury. LDA is an elegant and “context sensitive” solution, but assumes linearity, which may or may not be valid in burn injury. The method employed here incorporates a sophisticated model selection process, including nonlinear classifiers such as Artificial Neural Networks.

*Pattern Analysis*. The analysis of clustering provides several layers of information. The independent pattern analysis performed above provides the ideal number of variables that are necessary to capture the injury response. Detailed analysis of cluster membership may reveal further physiological information; for instance, in this work we used changing cluster membership between the liver inlets (hepatic artery and portal vein) and outlet (vena cava) for any particular metabolite as an indicator of altered liver function due to burn injury.

*Variable Elimination and Model Selection*. For each model (classifier) type considered, the variable elimination task involves finding an optimum list of variables that maximizes the model’s prediction accuracy. N-fold cross-validation accuracies were used to estimate the real-world accuracy for the trained regression models [35,36]: data are separated into N subsets; model training and validation is performed N-times, and in each run one of the subsets is used only for validation and the rest for model training. N-fold cross validation significantly reduces variability in accuracy estimates due to uneven validation data selections as all data is reused for testing the model accuracy. Multiple repetitions of the n-fold cross validation ensure that the selection process for the subsets does not affect the results.

For a given set of variables, comparison of alternative regression models is a straightforward task that can be based on the cross-validation results. If the number of variables is preset, the variable elimination is also a straightforward, albeit computationally intensive procedure: this problem is a combinatorial-optimization problem, where a set number of variables are chosen to maximize cross validation accuracy. In this work, we employed the variable selection algorithm in WEKA which employs a genetic algorithm to select the best variables to maximize the cross-validation accuracy for each classifier. This was followed by a ranking subroutine (Best-first algorithm in WEKA, a greedy step-climbing algorithm augmented with backtracking facility) which was used to select only the preset number of variables. 

However, the number of variables is typically a confounding factor. Briefly, the training accuracy improves in regression as the number of variables/free parameters increases, but this results in overtraining, such that the model is not valid for real data (*i.e*., the model is not *generalizable*), hence cross-validation accuracy will decrease after a certain point. The straightforward approach is to evaluate the cross-validation accuracy as a function of the number of variables used in the model, and choose the minimum number of variables that provide a high cross-validated accuracy, but this process is a very computationally intensive task since the variable elimination described above has to be repeated for each set number of variables, and this process has to be repeated for all possible models. Further, there are often multiple good solutions that have very similar accuracies. Therefore, in choosing the best combination of model, variables, and accuracy, determination of the proper weights of these competing objectives becomes a subjective decision.

By comparison, the number of independent patterns identified as described above, provides a simple criterion that can be determined through an objective and quick process. This approach also avoids issues during selection of the best regression model (*i.e.*, the decision of which model to use for the index of hypermetabolism), since number of variables employed by each model becomes an *a priori* set quantity that is equal for all tested alternatives. 

### 3.2. Methods

*Data Preprocessing*. The data per rat include metabolite concentrations of each of the PV, HA, and hepatic veins, and metabolic fluxes across the liver on day 4 post-burn. To eliminate outliers, variables with values beyond the median ± 2 × interquartile range for the group were considered as missing [37]. Since individual rat data are used in the construction of a hypermetabolism index, rats with >30% missing extracellular metabolite measurements were removed from the experimental dataset. Overall, 7 out of 12, 7/12, and 7/13 rat datasets were retained for the sham, 20% and 40% TBSA burn conditions, respectively. The missing values were replaced by the median of the measurements of that group. This resulted in a data matrix of 165 measurements on each of 21 animals.

*Analysis*. To identify major patterns in the dose response to burn, k-means clustering was performed in MATLAB. The average of each variable was calculated for each burn group (sham, 20%, 40%). Each variable was normalized to [−1 1] interval. The Euclidian distance was used as the clustering performance which provided better silhouette values compared to other distance measures (results not shown). Each clustering was run with >10 replicates and that the cluster means and their membership remained similar was confirmed (results not shown). It was observed that the changes in the cluster centroids were negligible between clustering runs, an indicator that the number of clusters was well chosen and cluster separation achieved was close to ideal. The centroid of each cluster was identified as the dose-response pattern.

Training, cross-validation, and selection of regression models were performed in WEKA data mining software [36]. The analysis was performed on per-rat data. The following classifiers were tested: Linear Regression (LR), normalized gaussian Radial Basis Function Network (RBFN), Neural Network (NN) (multilayer perceptron), Sequential Minimal Optimization algorithm for training a support vector Regression model (SMOR), M5P Decision Tree (M5P-DT), Decision Table (DT), M5 Rules (M5-R). For each method, variable selection was performed as described above. The accuracy of each regression model was then evaluated via five 10-fold cross validations, where all randomizable variables in the model, as well as the partitioning of data into 10 folds, were randomized. 

## 4. Results

**Figure 2:**
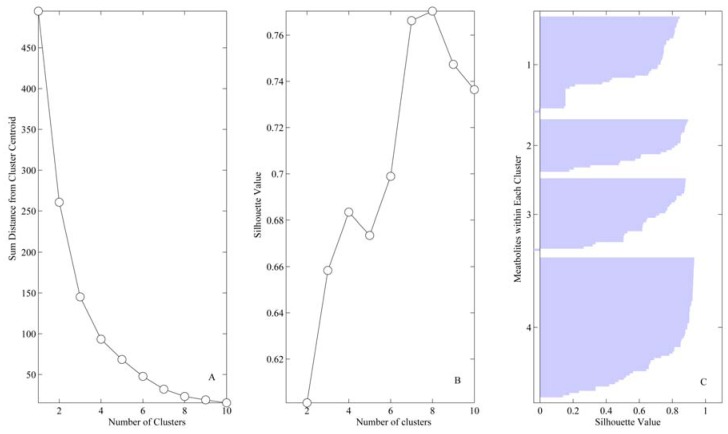
Cluster analysis indicates four distinct patterns present in metabolic profiles: (**A**) Number of clusters *vs.* sum distance from cluster centroid. (**B**) Number of clusters *vs.* silhouette value. (**C**) Silhouette values for each metabolite within the four clusters identified; a value of 1 indicates the metabolite profile is identical to the cluster centroid; −1 indicates the metabolite displays a perfect inverse profile.

**Figure 3:**
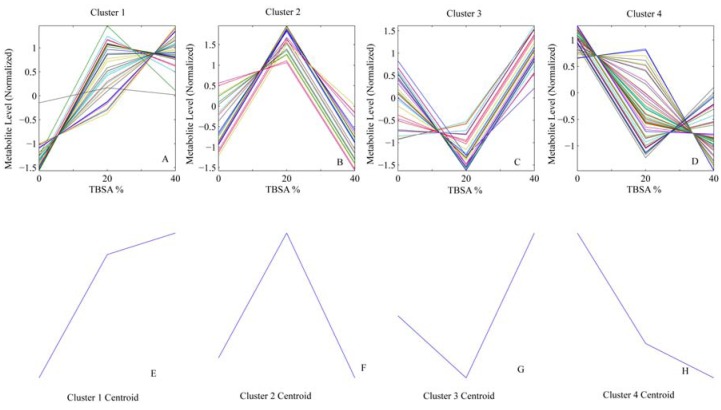
Identified Clusters. (**A**–**D**) Profile of metabolites classified within clusters 1–4. (**E**–**H**) Centroids (patterns) for each cluster.

*Clustering*. Analysis of the mean silhouette results demonstrated that increase from four to six cluster increased clusters separation only marginally (2.2%), hence four was chosen as the optimum number (Figure 2). Figure 3 displays the results of clustering along with the centroids of each cluster as a function of burn injury degree. Table 1 lists all the variables included in the analysis, their averages for each burn group, and the cluster membership for each variable.

**Table 1:** Variables included in analysis and cluster membership.

Variable	Vessel	Cluster	Sham (median ± IQR)	20%(median ± IQR)	40%(median ± IQR)
Arginine	HA	1	110.51 ± 10.239	182.13 ± 83.525	181.24 ± 14.245
Creatinine (Cre)	HA	1	0.2 ± 0	0.2 ± 0.05	0.2 ± 0
Cysteine	HA	1	15.76 ± 6.57	21.36 ± 0.92	25.55 ± 1.524
Fraction of Carboxyhemoglobin (FCOHb)	HA	1	7.9 ± 0.35	8.3 ± 1.5	8.5 ± 0.8
Fraction of Methlyated hemoglobin (Fmeth)	HA	1	0 ± 0	0 ± 0	0 ± 0
Low Density Lipoprotein (LDL)	HA	1	18 ± 14	22 ± 6	38 ± 8.75
Ornithine	HA	1	98.84 ± 22.359	117.73 ± 2.414	139.78 ± 12.129
Total Bilirubin (TBIL)	HA	1	0.3 ± 0.075	0.3 ± 0	0.3 ± 0.1
Total cholesterol/HDL ratio (TC/H)	HA	1	2.1 ± 0.1	2.2 ± 0.3	2.2 ± 0.05
Arginine	PV	1	113.3 ± 104.288	227.17 ± 42.862	223.04 ± 5.409
Aspartate Amino Transferase (AST)	PV	1	74 ± 16.5	83 ± 32.5	88 ± 27.5
AST/ALT	PV	1	1.73 ± 0.599	1.91 ± 0.82	2.1 ± 0.893
Cholesterol	PV	1	56 ± 24.5	74.5 ± 21.75	79 ± 10
Creatinine (Cre)	PV	1	0.2 ± 0	0.2 ± 0.05	0.2 ± 0
Cysteine	PV	1	14.89 ± 2.658	16.46 ± 1.543	16.55 ± 1.155
Fraction of Carboxyhemoglobin (FCOHb)	PV	1	7.2 ± 0.8	8.1 ± 1	7.7 ± 0.75
Flow Rate	PV	1	9 ± 9.226	20.5 ± 10.2	22 ± 6.5
Fraction of Methlyated hemoglobin (Fmeth)	PV	1	0 ± 0	0 ± 0.05	0 ± 0.1
Isoleucine	PV	1	91.03 ± 34.218	100.74 ± 12.663	99.65 ± 3.513
Low Density Lipoprotein (LDL)	PV	1	17 ± 6	30 ± 6	42.5 ± 9.5
Leucine	PV	1	243.09 ± 67.215	308.18 ± 25.007	336.71 ± 23.168
Lysine	PV	1	215.96 ± 92.604	234.02 ± 21.353	269.19 ± 20.227
Phenylalanine	PV	1	58.35 ± 9.465	65.69 ± 4.891	66.63 ± 7.422
Total Bilirubin (TBIL)	PV	1	0.3 ± 0.05	0.3 ± 0.05	0.3 ± 0.05
Total cholesterol/HDL ratio (TC/H)	PV	1	1.9 ± 0.1	2 ± 0.1	2.3 ± 0.05
Total CO2	PV	1	20 ± 4.5	23 ± 0	23 ± 3.5
Threonine	PV	1	175.33 ± 61.707	219.96 ± 60.936	228.44 ± 50.882
Tyrosine	PV	1	62.95 ± 8.409	73.73 ± 6.036	78.49 ± 8.038
Valine	PV	1	176.6 ± 62.552	206.26 ± 19.143	206.03 ± 4.825
Arginine	SHVC	1	62.27 ± 2.077	211.01 ± 23.971	206.12 ± 37.934
AST/ALT	SHVC	1	2.03 ± 0.786	2.41 ± 1.036	2.36 ± 0.913
Blood Urea Nitrogen (BUN)	SHVC	1	13 ± 2	15.5 ± 1.5	17.5 ± 2.5
Cholesterol	SHVC	1	49 ± 10	55 ± 22	74 ± 7
Creatinine (Cre)	SHVC	1	0.2 ± 0	0.2 ± 0.05	0.2 ± 0
Cysteine	SHVC	1	12.07 ± 0.595	16.68 ± 2.042	18.37 ± 2.285
Flow Rate	SHVC	1	9.5 ± 9.255	20.9 ± 10.2	22.2 ± 6.7
Fraction of Methlyated hemoglobin (Fmeth)	SHVC	1	0.25 ± 0.25	0.3 ± 0.15	0.3 ± 0.3
Isoleucine	SHVC	1	79.1 ± 12.184	103.88 ± 12.065	103.42 ± 8.164
Potassium (K)	SHVC	1	3.2 ± 0.95	4.2 ± 0.95	4 ± 1.15
Low Density Lipoprotein (LDL)	SHVC	1	17 ± 10.25	23 ± 6	36 ± 0
Ornithine	SHVC	1	77.44 ± 19.421	89.36 ± 35.165	123.36 ± 14.992
Total Bilirubin (TBIL)	SHVC	1	0.3 ± 0.05	0.3 ± 0	0.3 ± 0.05
Total cholesterol/HDL ratio (TC/H)	SHVC	1	2.05 ± 0.2	2.1 ± 0.1	2.2 ± 0
Valine	SHVC	1	121.63 ± 54.408	204.83 ± 24.603	206.6 ± 21.909
AST/ALT	HA	2	2 ± 0.752	2.48 ± 1.04	2.07 ± 0.733
Chloride (Cl)	HA	2	109 ± 10.5	114 ± 19	109 ± 10.5
Fraction of Oxyhemoglobin (FO2Hb)	HA	2	91.2 ± 4.85	91.5 ± 2.15	84.8 ± 8.35
Sodium (Na)	HA	2	143 ± 6	153 ± 16.5	142 ± 9
Partial pressure of CO2 (pCO2)	HA	2	43.95 ± 4.9	51.3 ± 12.65	46.8 ± 1.4
pH	HA	2	7.27 ± 0.059	7.3 ± 0.034	7.27 ± 0.042
Partial Oxygen pressure (PO2)	HA	2	106.2 ± 19.85	117.3 ± 18.5	100.35 ± 35.525
Tyrosine	HA	2	79.81 ± 17.969	88.17 ± 6.785	78.64 ± 8.325
Acetoacetate	PV	2	115.35 ± 115.91	134.15 ± 91.278	50.81 ± 115.089
Alanine	PV	2	391.58 ± 10.724	452.9 ± 113.48	395.22 ± 95.64
Alanine Amino Transferase (ALT)	PV	2	42 ± 11	46 ± 4.5	42 ± 4.5
β-hydroxybutyrate	PV	2	81.41 ± 87.551	103.82 ± 64.453	88.47 ± 49.107
Blood Urea Nitrogen (BUN)	PV	2	12 ± 5.5	16 ± 2.5	14 ± 3.5
Fraction of Oxyhemoglobin (FO2Hb)	PV	2	73.6 ± 12.4	82.7 ± 18.75	73.7 ± 15.2
Glucose	PV	2	143 ± 126.5	183 ± 55.5	147 ± 48.5
Potassium (K)	PV	2	3.7 ± 1.25	4.1 ± 0.7	3.7 ± 0.6
Partial pressure of CO2 (pCO2)	PV	2	55.6 ± 3.05	60.55 ± 5.5	57.5 ± 2.3
pH	PV	2	7.28 ± 0.01	7.3 ± 0.077	7.26 ± 0.028
Acetoacetate	SHVC	2	126.21 ± 27.78	126.21 ± 13.89	30.96 ± 95.246
Aspartate Amino Transferase (AST)	SHVC	2	84 ± 16	85 ± 13	80 ± 31
Fraction of Oxyhemoglobin (FO2Hb)	SHVC	2	23.3 ± 5.55	28.7 ± 17.65	14.1 ± 14
Leucine	SHVC	2	218.34 ± 52.758	261.92 ± 91.466	210.42 ± 147.222
pH	SHVC	2	7.29 ± 0.02	7.32 ± 0.055	7.25 ± 0.021
Partial Oxygen pressure (PO2)	SHVC	2	27.2 ± 0.725	41.9 ± 15.6	15.1 ± 11.4
Alkaline phosphatase (ALP)	HA	3	192 ± 96.5	113 ± 21	194 ± 74
Aspartate Amino Transferase (ALT)	HA	3	49.5 ± 14.25	36 ± 7.5	39 ± 11.5
β-hydroxybutyrate	HA	3	183.61 ± 194.894	65.45 ± 39.132	165.2 ± 104.353
Blood Urea Nitrogen (BUN)	HA	3	12.5 ± 7	13 ± 1.5	17 ± 4.5
Calcium (Ca)	HA	3	8.5 ± 1.25	7 ± 1	8.8 ± 0.85
Cholesterol	HA	3	56.5 ± 14.75	58 ± 13	73 ± 21
Fraction of Deoxyhemoglobin in Total Hemoglobin (FHHb)	HA	3	0.3 ± 4.45	0.2 ± 0.4	0.3 ± 7.15
Glutamine	HA	3	406.45 ± 97.249	344.31 ± 47.021	414.34 ± 24.774
High Density Lipoprotein (HDL)	HA	3	33 ± 5	27 ± 18	36 ± 6.75
Histidine	HA	3	65.3 ± 0.404	61.03 ± 9.453	67.39 ± 8.07
Lactate	HA	3	0.84 ± 0.944	0.58 ± 0.942	3.47 ± 1.49
Lysine	HA	3	220.96 ± 66.163	181.93 ± 25.467	224.74 ± 13.38
Phenylalanine	HA	3	65.09 ± 7.564	63.2 ± 3.598	67.17 ± 1.202
Total CO2	HA	3	21 ± 1.5	19 ± 1.5	25 ± 3
Total Protein (TP)	HA	3	5.3 ± 0.5	3.7 ± 0.7	4.7 ± 0.3
Alkaline phosphatase (ALP)	PV	3	174 ± 69	151 ± 33.5	184 ± 78
Chloride (Cl)	PV	3	110 ± 14.5	109 ± 13	112 ± 7
Fraction of Deoxyhemoglobin in Total Hemoglobin (FHHb)	PV	3	19.2 ± 13.25	9.2 ± 19.7	18.6 ± 15.85
High Density Lipoprotein (HDL)	PV	3	36 ± 12	33 ± 4.5	35.5 ± 2.5
Histidine	PV	3	140.94 ± 16.226	120.93 ± 9.821	147.78 ± 19.839
Lactate	PV	3	1.07 ± 0.095	0.68 ± 0.632	4.38 ± 1.986
Sodium (Na)	PV	3	146 ± 6.5	146 ± 12.5	149 ± 2.5
Ornithine	PV	3	119.81 ± 4.751	107.16 ± 6.154	120.82 ± 17.96
Proline	PV	3	161.22 ± 18.151	162.06 ± 8.013	171.2 ± 35.127
Fraction of Deoxyhemoglobin in Total Hemoglobin (FHHb)	SHVC	3	71.1 ± 6	59 ± 15.8	80 ± 13.45
High Density Lipoprotein (HDL)	SHVC	3	26.5 ± 6	24 ± 5	33 ± 0
Histidine	SHVC	3	53.01 ± 9.45	52.2 ± 18.937	61.6 ± 6.314
Lactate	SHVC	3	1 ± 0.34	0.74 ± 0.141	3.81 ± 2.135
Lysine	SHVC	3	205.05 ± 79.978	197.38 ± 80.877	210.04 ± 29.84
Sodium (Na)	SHVC	3	148 ± 7	148 ± 12.5	149 ± 7.5
Partial pressure of CO2 (pCO2)	SHVC	3	55.2 ± 6.9	51.3 ± 3.75	67.4 ± 7
Total CO2	SHVC	3	22 ± 4	22 ± 2	25 ± 0.75
Tyrosine	SHVC	3	61.88 ± 6.68	61.29 ± 9.773	68.37 ± 7.3
Acetoacetate	HA	4	116.29 ± 22.819	110.34 ± 47.623	70.65 ± 31.749
Alanine	HA	4	348.27 ± 41.954	297.32 ± 22.715	281.4 ± 33.083
Albumin	HA	4	1.95 ± 0.175	1.2 ± 0.3	1.3 ± 0.25
Alanine Amino Transferase (ALT)	HA	4	49.5 ± 14.25	36 ± 7.5	39 ± 11.5
Ammonia	HA	4	49.7 ± 4.527	37.3 ± 4.081	23.1 ± 4.616
Asparagine	HA	4	41.98 ± 5.488	34.51 ± 3.945	32.73 ± 6.58
Aspartate	HA	4	11.83 ± 1.549	9.25 ± 5.852	10.16 ± 2.637
Hemoglobin (%)	HA	4	12.4 ± 1.35	12 ± 2.4	10.4 ± 1.05
Flow Rate	HA	4	0.65 ± 0.146	0.5 ± 0.069	0.5 ± 0.426
Glucose	HA	4	169 ± 87	134 ± 46	146.5 ± 16
Glutamate	HA	4	76.85 ± 6.662	54.15 ± 19.137	46.84 ± 17.453
Glycine	HA	4	216.75 ± 16.195	184.88 ± 25.974	163.76 ± 19.223
Hematocrit	HA	4	36 ± 3.5	35 ± 7	31 ± 3
Isoleucine	HA	4	113.82 ± 47.423	112.05 ± 19.396	108.01 ± 32.529
Potassium (K)	HA	4	4.25 ± 2.15	4.2 ± 1.45	3.9 ± 0.75
Leucine	HA	4	369.7 ± 174.817	275.1 ± 32.956	285.2 ± 87.999
Methionine	HA	4	52.33 ± 11.417	40.59 ± 8.321	38.75 ± 9.047
Proline	HA	4	164.52 ± 9.956	147.7 ± 27.492	147.69 ± 7.333
Serine	HA	4	223.83 ± 54.196	159.53 ± 12.872	169.06 ± 26.87
Triglycerides (TG)	HA	4	62 ± 35.75	29 ± 7	26 ± 6
Threonine	HA	4	256.03 ± 36.785	229.07 ± 41.332	202.04 ± 15.094
Valine	HA	4	234.98 ± 107.828	184.35 ± 36.559	178.74 ± 33.851
Very low density lipoprotein (VLDL)	HA	4	13 ± 0.5	6 ± 1	5 ± 0.5
Albumin	PV	4	2 ± 0.275	1.3 ± 0.35	1.3 ± 0.2
Ammonia	PV	4	108.65 ± 61.525	43.74 ± 33.121	72.67 ± 10.709
Asparagine	PV	4	51.08 ± 6.886	32.09 ± 3.895	31.74 ± 0.837
Aspartate	PV	4	19.18 ± 11.097	12.7 ± 1.594	12.06 ± 2.288
Calcium (Ca)	PV	4	8.7 ± 2.25	8.3 ± 0.55	7.9 ± 1.65
Hemoglobin (%)	PV	4	14.6 ± 0.95	13 ± 1.75	12.8 ± 0.5
Glutamate	PV	4	63.16 ± 13.278	42.22 ± 11.148	48.44 ± 15.319
Glutamine	PV	4	279.12 ± 59.911	253.05 ± 70.109	250.29 ± 33.243
Glycine	PV	4	273.49 ± 48.285	222.25 ± 33.939	204.59 ± 46.681
Hematocrit	PV	4	43 ± 3.5	40 ± 5	38 ± 2.5
Methionine	PV	4	44.77 ± 4.889	44.17 ± 4.749	43.82 ± 3.209
Partial Oxygen pressure (PO2)	PV	4	69.9 ± 11.75	54.2 ± 8.1	62.9 ± 15.65
Serine	PV	4	202.59 ± 14.805	148.88 ± 30.334	156.79 ± 14.701
Triglycerides (TG)	PV	4	52 ± 43	25.5 ± 11.75	23 ± 2
Total Protein (TP)	PV	4	5 ± 1.25	4.7 ± 0.9	4.8 ± 0.85
Very low density lipoprotein (VLDL)	PV	4	13 ± 5	6 ± 1	5 ± 0.5
Alanine	SHVC	4	246.38 ± 53.891	156.72 ± 42.529	152.26 ± 14.02
Albumin	SHVC	4	1.9 ± 0.25	1.3 ± 0.25	1.2 ± 0.25
Alkaline phosphatase (ALP)	SHVC	4	166 ± 67.5	129 ± 21	147 ± 82
Alanine Amino Transferase (ALT)	SHVC	4	40 ± 15.5	38 ± 14	37 ± 5.5
Ammonia	SHVC	4	40.44 ± 2.622	21.86 ± 10.951	15.01 ± 9.875
Asparagine	SHVC	4	27.87 ± 1.556	20.1 ± 3.165	18.34 ± 0.097
Aspartate	SHVC	4	13.57 ± 1.938	10.37 ± 4.932	8.47 ± 0.817
β-hydroxybutyrate	SHVC	4	275.69 ± 457.311	257.28 ± 244.001	223.51 ± 174.944
Calcium (Ca)	SHVC	4	8.6 ± 2.5	7.9 ± 0.3	8 ± 1.5
Chloride (Cl)	SHVC	4	111 ± 14.5	110.5 ± 4.75	109 ± 11
Hemoglobin (%)	SHVC	4	12.75 ± 0.875	10.7 ± 1.75	11.6 ± 0.65
Fraction of Carboxyhemoglobin (FCOHb)	SHVC	4	290 ± 73.75	130 ± 24	176 ± 50
Glucose	SHVC	4	263 ± 73	186 ± 72.5	152 ± 36.25
Glutamate	SHVC	4	76.77 ± 43.277	49.62 ± 15.79	44.7 ± 19.093
Glutamine	SHVC	4	299.7 ± 56.737	254.82 ± 97.786	228.06 ± 93.284
Glycine	SHVC	4	192.45 ± 57.864	148.04 ± 8.773	117.74 ± 58.252
Hematocrit	SHVC	4	37.5 ± 2.5	31 ± 5	34 ± 1.5
Methionine	SHVC	4	38.47 ± 1.541	32.45 ± 2.444	34.55 ± 1.049
Phenylalanine	SHVC	4	54.68 ± 3.359	52.83 ± 15.86	49.57 ± 14.92
Proline	SHVC	4	137.82 ± 37.486	115.64 ± 6.032	113.16 ± 16.448
Serine	SHVC	4	166.82 ± 20.803	105.99 ± 12.727	92.92 ± 42.759
Triglycerides (TG)	SHVC	4	25 ± 28.5	20 ± 3	20 ± 0
Threonine	SHVC	4	198.18 ± 20.372	186.17 ± 59.461	157.26 ± 6.575
Total Protein (TP)	SHVC	4	4.9 ± 1.05	4.3 ± 0.45	4 ± 0.85
Very low density lipoprotein (VLDL)	SHVC	4	9.5 ± 4.5	5 ± 0	5 ± 0

*Identification of Major Patterns*. While there are four major patterns in the metabolic response to burn, they are not necessarily independent. For instance, clusters 1 and 4 apparently display the inverse of the same response. To identify the number of independent patterns, SVD Analysis was employed. This analysis revealed that two patterns explained 98.6% of the total variability observed, indicating that the remaining two patterns were explainable within a margin of error of <2%. The patterns highest weighted in SVD were clusters #2 and #4. It should be noted that the pattern selection here is based purely on overall trends, unlike the analysis of Yang *et al.* [34] which identifies critical time points of sudden changes in gene expression. Since the resolution of data for burn injury is limited (*i.e.*, we have only three levels of burn injury), it is not possible to employ a similar method to identify the degree of injury where the post-burn hypermetabolic response kicks in. 

*Analysis of Cluster Membership*. In general there were no strong trends observed in the variables comprising the clusters (Table 2). Cluster 2 had nearly equal membership from all vessels. Cluster 4 had the most variables (68 total). Albumin, asparagine, aspartate, glutamate, methionine, serine, hematocrit, triglycerides and VLDL concentrations systemically (*i.e.*, in all vessels observed) were selected to cluster 4, which displayed a general decreasing response with increasing burn. pH and FO_2_Hb were in cluster 2, which showed a peak at 20% burn. Lactate had increased systemically and was in cluster 3 with a sharp increase at 40% burn; HDL and histidine also belonged in this cluster systematically, although the sharp increase at maximum burn was not present. LDL and cysteine increased systemically, and were in cluster 3.

**Table 2:** Cluster membership as a function of vessel/flux.

Cluster	# variables in vessel/flux				
	Hepatic Artery	Portal Vein	Vena Cava	Liver Flux	Total
1	9	20	15	9	45
2	8	10	6	8	26
3	15	9	9	15	36
4	23	16	25	23	68
Total	55	55	55	5	

To analyze the role of the liver in the dose response to burn injury, the variables that were selected to the same cluster in the liver inlet (PV and HA) but different outlet (SHVC) were identified. FCOHb (HA and PV in cluster 1, SHVC in cluster 4), pCO2 (2/3), ALP (3/4) were the three variables identified.

### 4.1. Model Selection and Index of Burn Injury Severity

The variable elimination process was performed with each model with the number of variables set at two. The two best models were the M5-Rules and NN, with very similar cross validation accuracies (Table 3). The formula for the index of burn injury severity developed via the NN model is:


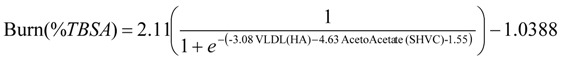


**Table 3:** Comparison of tested models.

	Average Relative Accuracy	Variable 1	Variable 2
RBF	46.06 ± 4.59	Ammonia (HA)	Ornithine (SHVC)
DT	70.54 ± 7.83	Asparagine (SHVC)	ALB (HA)
M5P-DT	72.36 ± 1.03	ALB (HA)	Ammonia (HA)
LR	73.28 ± 0.55	VLDL (HA)	Acetoacetate (SHVC)
SMOR	75.92 ± 2.20	VLDL (HA)	Acetoacetate (SHVC)
M5-R	87.82 ± 1.31	VLDL (HA)	Asparagine (SHVC)
NN	87.89 ± 2.79	VLDL (HA)	Acetoacetate (SHVC)

An interesting observation was that very low density lipoprotein (VLDL) (cluster #4) was a common choice in all the high-accuracy models. The acetoacetate level in the SHVC was the second selection in the NN model, whereas SHVC asparagine concentration was selected in the M5P-R model. The top two regression models, NN and M5P-R, had nearly identical ~88% cross validation accuracy, which has value as a diagnostic index, but with significant room for improvement.

The effects of using additional variables in the index were evaluated for NN and M5P-R (Table 4). The NN model was selected as the regression model of choice since accuracy increased significantly (23%) with up to 4 variables. By contrast, the accuracy of the M5P-R model was slightly increased with a third variable, but later decreased with addition of variables (it should be noted this is indeed the expected situation with proper cross-validation, which can account for over training; without validation data the accuracy would have increased monotonously with addition of new variables). The predictions of the NN model on a case-by-case basis are displayed in Table 5. However, note that these are the results of training on the full set of data, hence the overall accuracy is significantly higher than it would be expected in an actual application, which is what the cross validation results in Table 3 report. 

**Table 4:** Effect of number of variables on regression accuracy.

Method	Measure	2 Variables	3 Variables	4 Variables	5 Variables
NN	Average Relative Accuracy	87.89 ± 2.789	89.46 ± 1.379	90.69 ± 1.508	89.18 ± 1.552
	Selected Variables	VLDL (HA), Acetoacetate (SHVC)	VLDL (HA), Acetoacetate (SHVC), pO2 (SHVC)	VLDL (SHVC), Acetoacetate (SHVC), pO2 (SHVC), CO2 (HA)	VLDL (SHVC), Acetoacetate (SHVC), pO2 (SHVC), Asparagine (SHVC), CO2 (HA)
M5-R	Average Relative Accuracy	87.82 ± 1.312	88.71 ± 0.983	87.66 ± 1.398	87.11 ± 1.038
	Selected Variables	VLDL (HA), Asparagine (SHVC)	Albumin (HA), Asparagine (SHVC), BUN (PV)	VLDL (HA), Acetoacetate (SHVC), Albumin (HA), pCO_2_(SHVC)	VLDL (HA), Acetoacetate (SHVC), Albumin (HA), pCO_2_(SHVC) Asparagine (SHVC)

**Table 5:** Predictions of neural network model (Training on all data).

Instance	Burn % (actual)	Predictions			
		2 Variable Model (%burn)	3 Variable Model (%burn)	4 Variable Model (%burn)	5 Variable Model (%burn)
1	0	0.132	0.324	1.35	1.271
2	0	−0.281	−0.373	−0.423	−0.320
3	0	3.003	0.109	0.05	−0.195
4	0	−0.614	0.415	−0.632	−0.52 0a
5	0	0.309	−0.713	−0.011	−0.013
6	0	−0.416	−0.213	−0.451	−0.635
7	0	−0.281	0.079	−0.301	−0.229
8	20	19.048	19.876	20.654	20.528
9	20	19.048	20.171	18.383	18.014
10	20	19.048	19.585	19.72	19.590
11	20	19.048	19.585	19.72	19.59 0
12	20	25.415	19.686	19.993	19.715
13	20	19.048	19.510	18.22	17.947
14	20	20.255	20.757	20.651	17.947
15	40	40.709	39.911	39.795	39.246
16	40	40.957	40.268	40.919	40.870
17	40	40.709	40.091	40.045	39.633
18	40	41.238	39.938	39.866	39.709
19	40	41.221	38.822	39.612	39.620
20	40	39.865	39.757	38.984	38.333
21	40	36.667	39.739	39.708	39.546
*Relative Absolute Error*		*8.49%*	*2.54%*	*4.10%*	*5.04%*

Finally, since selection of acetoacetate is highly significant as an indicator of mitochondrial redox potential, we also tested the prediction success when acetoacetate/β-hydroxybutyrate ratio (a commonly used predictor of redox potential) is used [38,39]. Briefly, we repeated the variable selection/training algorithm for the following set: acetoacetate/β-hydroxybutyrate ratio (SHVC), acetoacetate/β-hydroxybutyrate ratio (HA), VLDL (SHVC), pO_2_ (SHVC), CO_2_ (HA), as well as acetoacetate/β-hydroxybutyrate ratio (SHVC) alone with NN and M5-R models. On its own, the acetoacetate/β-hydroxybutyrate ratio was extremely unsuccessful in predicting %TBSA, (average relative errors of 110.87 ± 7.444 and 110.26 ± 3.063 with NN and M5-R, respectively). Inclusion of other variables marginally improved the results but nowhere near the other results in Table 4: The average relative errors were 76.26 ± 15.887 and 64.55 ± 10.283 for NN and M5-R, respectively.

## 5. Discussion and Conclusions

The most important finding of this work is the strong correlation between VLDL levels and the extent of burn injury. Prior studies indicate that in burn trauma, VLDL secretion is impaired [40]. This is consistent with our observations that for increasing burn, there was a decrease in systemic VLDL. Interestingly, VLDL, LDL, HDL and TG are four of the relatively few variables that were systemically altered (*i.e.*, belonged in the same cluster independent of the measured blood vessel). However, the model developed here suggests that VLDL is the most preferred variable for building a predictor of severity of burn injury; ahead of other potential metabolic targets such as hyperglycemia which is correlated to insulin resistance [9], alanine/glucose (a systemic cycle for conversion of skeletal muscle to glucose during hypermetabolism), or even glutamine and arginine, which tend to be depleted after burn injury [5,41,42]. Of note here is that as clustering analysis shows, other potential variables identified in Table 4 demonstrate altering (*i.e.*, belonging to different clusters based on measured vessel) injury dose-response profiles; therefore, predictions based on VLDL are least likely to be affected by tissue specific variations. Very likely, the fact that VLDL decrease is systemic also renders it a more accurate indicator of hypermetabolism.

The second variable of interest, which was selected commonly for most regression models (including M5P-R with 3 or more variables) is acetoacetate (SHVC), which in the context of burn is most important as the precursor for β-hydroxybutyrate. While ketone bodies are not a subject of intense focus in hypermetabolism, it was previously suggested that the acetoacetate/β-hydroxybutyrate ratio reflects the mitochondrial redox potential in the liver [43,44], and in burn patients, a decrease of plasma acetoacetate to β-hydroxybutyrate ratio indicates mitochondrial dysfunction and correlates with developing multiple organ dysfunction [45]. Our rat data show a significant decrease in acetoacetate in the 40% TBSA burn group (Table 1). β-hydroxybutyrate was also decreased, although to a lesser extent, such that the acetoacetate to β-hydroxybutyrate ratio was decreased as well. In general, the decrease in total ketone bodies is regarded as an indicator of a limitation in oxidative phosphorylation after injury [46]. Concomitantly, other variables linked to mitochondrial activity or respiration, such as venous CO_2_, were also selected as additional injury indicators by the Neural Network model. It should be noted however, that the acetoacetate/β-hydroxybutyrate ratio by itself was not a particularly useful indicator of extent of burn injury. Further, replacing acetoacetate by the ratio severely decreased the accuracy achievable, with 100% error range, indicating that since the ratio was unsuccessful both on its own and when replacing acetoacetate concentrations, this reduction in accuracy is likely to be at least partially due to amplification or measurement noise. Note that β-hydroxybutyrate was one of the measurements with highest standard deviations relative to group means.

A third variable that was commonly selected was asparagine. Asparagine/aspartate is one of the cycles that carry amino acids from the muscle tissue to the liver during injury and was previously reported to be altered in rat models of burn injury [47]. While asparagine was able to replace acetoacetate in the M5P-R model, for the NN model that was ultimately more successful and could exceed 90% accuracy acetoacetate proved a better variable to include in the index.

It is worth noting that VLDL (SHVC) is not significantly different between the 20% and 40% burn groups, but is significantly reduced compared to the sham group. It can be argued that the ability to differentiate between sham and burn provided by VLDL complements acetoacetate (SHVC), which is different for the 40% burn group, but similar for the sham and 20% burn groups. The interesting note here is that neither of these variables is simply directly correlated to extent of burn, hence use of multiple variables is necessary. This supports our previous findings [21,48] that there are significantly different responses observed between the 20% and 40% burn groups, either because the response at 20% TBSA is significantly less, or possibly as the 20% group is displaying a switch from hypermetabolism to a healing/normal phase as early as 4 days after burn injury. This response is likely the reason that a nonlinear model, such as the multi-layer perceptron, has higher accuracy than other linear models we tested in this study. This result may also justify why hypermetabolism diagnosis is still best based on clinical observations because this ad hoc method allows the physician to account for the nonlinear behavior based on past experience.

Using the 2-variable minimum identified by clustering, an index of burn injury severity was developed. The cross-validated accuracies of the best regression model, artificial Neural Network, was 88%. We also tested the inclusion of additional variables into the regression model. As displayed in Table 4, the accuracy of the multilayer perceptron model could be increased to up to 91% with the addition of arterial total CO_2_ (cluster 3) and venous oxygen (cluster 2). While the NN therefore did not include any variable from cluster 1, since cluster 4 displays the inverse dose-response to cluster 1 very closely, this confirms the previous analysis that 2 clusters are sufficient in capturing most of the metabolic response to increased burn injury.

From an application perspective, VLDL is in the same cluster systemically; hence point of measurement is unlikely to affect results significantly (repetition of 2-variable NN model with SHVC VLDL use led to only one rat in the sham burn group being significantly misclassified as 20% burn animal). Acetoacetate also displays a generally decreasing trend in all vessels. It should be noted that for an actual clinical index, ideally all measured variables will be systemically in the same cluster, as well as practical to measure. Most variables meet the criteria selected in the regression models. It is also likely possible to construct a regression model to predict unsuitable metabolites from more easy-to-measure ones, which we have not investigated here; a proper study of such an approach would require repetition of the experiments with tail-vein blood sample data complementing the HA, PV and SHVC samples to test if these less invasive samples correlate accurately to the data in this work.

To our knowledge this is the first attempt at creating a quantitative index of burn injury severity; however, it is important to realize the limitations in clinical applications. As the animals were not subjected to any intervention following burn injury, there were no potential confounding effects from nutritional supplementation, which may not be the case in human patients. Obviously, differences between rat and human metabolism, as well as effects of age and gender differences on the hypermetabolic response [48], will have to be considered for the development of a clinically applicable index.

These indicators of burn injury may provide insight and clues to new metabolic targets for therapy. In addition, the development of a quantitative score to identify the degree of hypermetabolism can ultimately provide a practical way to measure the patient response to injury and treatment. As the current diagnostic criteria are based purely on clinical observations, patient-to-patient variation in metabolism introduces a significant and undesirable degree of uncertainty in the care of the burn patient that could be avoided by such a quantitative index.
